# Attachment styles and their association with aggression, hostility, and anger in Lebanese adolescents: a national study

**DOI:** 10.1186/s40359-022-00813-9

**Published:** 2022-04-22

**Authors:** Elise Maalouf, Pascale Salameh, Chadia Haddad, Hala Sacre, Souheil Hallit, Sahar Obeid

**Affiliations:** 1 Department of Life and Science, Paris Est University, Paris, Lebanon; 2 School of Medicine, Lebanese American University, Byblos, Lebanon; 3 INSPECT-LB (Institut National de Santé Publique, d’Épidémiologie Clinique Et de Toxicologie-Liban), Beirut, Lebanon; 4 Department of Primary Care and Population Health, University of Nicosia Medical School, 2417 Nicosia, Cyprus; 5 Faculty of Pharmacy, Lebanese University, Hadat, Lebanon; 6 School of Medicine and Medical Sciences, Holy Spirit University of Kaslik, P.O. Box 446, Jounieh, Lebanon; 7 Psychology Department, College of Humanities, Effat University, Jeddah, 21478 Saudi Arabia; 8 Research Department, Psychiatric Hospital of the Cross, Jal Eddib, Lebanon; 9 Social and Education Sciences Department, School of Arts and Sciences, Lebanese American University, Jbeil, Lebanon

**Keywords:** Attachment styles, Physical aggression, Verbal aggression, Hostility, Anger

## Abstract

**Background:**

The idea that attachment styles can affect the level of anger in an individual educes a reason why people develop anger issues and behavioral problems in adolescence that escalate into adulthood. Lebanon suffers from a shortage of data pertaining to insecure attachment styles and the affective and cognitive aspects of anger and behavioral anger expression among the Lebanese youth population. This study aimed to investigate the association between attachment dimensions and anger expression (trait anger, hostility, physical aggression, and verbal aggression) among a sample of Lebanese adolescent participants.

**Methods:**

This cross-sectional study was performed between January and May 2019 among 1810 Lebanese high-school students aged 12–18 and used two validated measures, the Adolescent-Relationship Questionnaire (A-RQ) and The Buss-Perry Aggression Questionnaire (BPAQ). The A-RQ assessed attachment behaviors, while the BPAQ evaluated aggression.

**Results:**

Higher fearful and dismissing attachment styles, and higher physical activity index were significantly associated with higher physical and verbal aggression. A higher fearful attachment style was significantly associated with more anger. A higher secure attachment style was significantly associated with less anger. Higher preoccupied and dismissing attachment styles were significantly associated with higher hostility.

**Conclusion:**

Our findings revealed a significant relationship between both insecure attachment dimensions and the tripartite model of anger expression. This study adds to the anger literature by providing a more informed understanding of how variations in anger expression are linked to the processing of interpersonal interactions, which are the hidden facets of attachment systems.

## Background

Anger is a normal and common healthy emotion. It is a fully adaptive response to frustrating or difficult situations. However, losing control of it may be harmful to both emotional and physical health. Anger only becomes an issue when it is overly shown and begins interfering with everyday functioning and interpersonal relationships. In such cases, it may lead to detrimental consequences frequently connected with hateful thoughts, physiological wakening, and maladaptive behaviors [1]. Individuals who perceive a threat or danger to their emotional or physical well-being, their resources, such as time, energy, properties, and wealth, or their loved ones, experience anger arousal. It acts on a continuum from mild frustration to absolute fury; therefore, the intensity of anger determines the behavioral anger response [2]. Buss and Warren suggested a tripartite model of anger expression, comprising three definite but related facets: emotion/affect (trait anger: propensity to exhibit angry feelings), cognition (hostility: a cognitive facet of aggression characterized by feelings of resentment and malevolence towards others), and behavior such as physical and verbal aggression [3].

The most prevalent concerns expressed by parents, teachers, and educators are anger and aggressiveness in adolescents [4].

To begin with, teens and emerging young adults between 15 and 19 are still in the process of learning about their intense and changing emotions. They do not entirely grasp the physical and mental takeover that may arise while they are upset. As children age, higher suppression of their frustration can be seen, as they no longer feel that more emotions can be displayed. Apart from this consideration, an overt display of anger can be viewed by students as less permissible or socially unacceptable [5]*.* Essentially, when teenagers are discouraged from undertaking physical or social tasks, or when they are in the situation of being attacked on their identities, roles, or social status, they are offended [6]*.* For teenagers, anger is a secondary emotion since it frequently hides other underlying difficulties such as sadness, pain, fear, and humiliation. Teenagers are more likely to show anger if boys and younger compared to girls and older teens [5]*.* Besides, as opposed to teenage girls who respond verbally or non-violently to anger, adolescent males react to anger with overtly offensive reactions and physical assaults on objects [7]*.* A frequent trigger of anger responses in adolescence are parent–child clashes, which involve largely small fights, as teenagers are constantly attempting to shift from dependency to self-reliance [8, 9]*.* According to the idea of multifinality in developmental psychopathology, extremely prone to anger infants will not all follow harmful developmental paths. Therefore, several circumstances might lead to adolescent anger and resistance. Multiple elements, at various levels, have been found as interacting with early anger proneness, including facets of the parent–child attachment, family situation, appreciation and affection among family members, the household crowding, early stress, and numerous social and contextual factors such as regular physical activity and smoking behaviors [10–13].

The quality of the connection that develops between the adolescent and the parent can be a notably essential component with an everlasting impact on the psychological and behavioral states of the adolescent. Indeed, soothing and protective caregiver-child interaction promotes the proper emotional self-regulation capacities in the developing child [14]. Therefore, the attachment theory could be a helpful theoretical framework to foster comprehension of adult disparities in emotional and behavioral coping abilities.

Attachment theory is becoming profoundly vital when studying interpersonal behavior and individual differences in emotion regulatory processes in adulthood [15, 16]. According to John Bowlby's attachment theory (1969), the attachment system is a genetically programmed system, with a biological basis, designated to protect the baby due to his insufficient autonomy to ensure his essential survival needs. The secure core concept (response to threat and stress) was at the center of attachment theory [17]. Bowlby noted that emotional attachment to parents has a substantial impact on relationships throughout life and that this long-term bond between a parent and child is also a component in the growing person's ability to deal with stressful or demanding conditions [18]. Moreover, children gain the capacity and flexibility to seek safety and care when facing physical dangers or negative emotions and exercise communication and resilience when away from the caregiver's direct vicinity [14].

Studies have demonstrated that parental and child influences play a role in establishing and sustaining children externalizing behaviors [19]. During their development period, young adults run into many interpersonal difficulties. Young adulthood is a period in which cognitive control is particularly susceptible to negative emotional factors [20], as they are confronted with issues related to identity, separation, relationships, and purpose. Their relationship with their parents also shifts as they become more and more independent. Moreover, parenting contributes significantly to self-control in adolescents aged 10 to 22. It also implies that adolescent self-control has a considerable delayed effect on future parenting [21]. Hence, the association between parenting styles and self-control should be highlighted. Theorists agree that parenting is an essential factor associated with individual differences in self-control [22, 23]. More specifically, prior studies have found that positive parenting (e.g., monitoring, consistent discipline, parental warmth and support, positive control, authoritative parenting) and a solid parent–child relationship (e.g., secure attachment, close parent–child bonding, high-quality relationship) are related to better self-control. Conversely, negative parenting (e.g., inconsistent discipline, harsh parenting, coercive parenting, physical punishment, negative control, authoritarian parenting) and a weak parent–child relationship are associated with lower self-control in early and middle childhood [8, 9, 24]. However, studies extending this work to adolescence yielded mixed findings. While some studies have reported robust cross-sectional and longitudinal associations between parenting and self-control throughout adolescence, others have reported only significant concurrent associations [8, 9, 25].

Attachment has often been the topic of several psychological investigations and the cornerstone for therapeutic procedures. It is predicated on a concept that describes how emotions are managed and how life events are perceived in relation to others. In clinical and research settings, two approaches are being used. First, individual attachment is evaluated in terms of attachment dimensions, with attachment security, avoidance, and anxiety being prioritized [26]. Next, indications from these dimensions are merged to form various patterns. When it comes to assessing attachment variables, adolescents are described as having four attachment styles: Secure, Anxious-preoccupied, Dismissive-avoidant, and Fearful-avoidant [27]. Each style is associated with different dynamics. It is argued that a secure connection is one in which the child's needs are constantly fulfilled by their parent; the child will then evolve a sense of self-respect and become more open to trusting others, which will transcend into adulthood. The child would then accept more and be more comfortable in expressing their desires and feelings candidly. On the flip side, an insecure attachment emerges from the incongruity of the caregiver's reaction to the essential needs of the infant or child. More particularly, individuals with anxious-preoccupied attachment types appear to have low self-esteem and a more positive perception of others. They crave protection and affection from others. A dismissive-avoidant type of attachment is displayed by those who have a positive view of self and a negative view of others. Indeed, due to avoidant childhood attachment, they treasure their independence and may get anxious when someone approaches them too closely. It is critical for them to feel self-sufficient. For the fearful type, both perceptions of self and others are negative; the person does not feel loveable and assumes that others will be discarded and untrustworthy [27]. In numerous studies, researchers popularized the image of caregivers as the "secure basis" for infants and the four attachment styles. Consequently, this concept will be prioritized in our study when analyzing attachment in adolescents. A growing body of research advocates the relation between attachment and the way of communicating anger; in comparison to healthy controlled anger expression, cases with insecure attachment were associated with high hostility [28] and overall aggression [29, 30], the most common and potentially destructive manifestations of anger.

In particular, Bowlby posited that the internal working models of relationships emerge from early attachment to caregivers and play a significant role in the experience of anger. He specifically emphasized that the state of the parent–child relationship had a considerable influence on an individual's emotional development. If the relationship is going well, a sense of security develops, and anger is expressed healthily; if the connection is constantly challenged, skewed emotional reactions, such as excessive or irrational anger, emerge [18]. Persistent warnings of abandonment and rejection by an attachment figure, especially, are thought to elicit extreme anger responses, which are then utilized to prevent the attachment figure from carrying out the threat. The attachment of adolescents from unfavorable caregiving environments, such as residential care (RC) or late adoption (LA), has been compared with that of community peers raised by their biological families [31]. Only institutionalized adolescents demonstrated more insecure and disordered patterns than both late-adopted and community peers in a variety of attachment-related domains where RC and LA struggled throughout childhood [31]. They also displayed more parental anger and disrespect and a lower convergence between friends and family interview (FFI) and the inventory of parent and peer attachment (IPPA) than the other two groups, suggesting the presence of a defensive need to downplay or reject adverse experiences and revealing the high levels of anger embodying these insecure (dismissive or preoccupied) interviews [31]. These findings support the fact that the steady qualities of adoption can promote security, especially if caregivers are responsive and sensitive to the child's needs, but the instability and many placements typical of residential care might be detrimental. In other words, assessing the link between attachment and anger expression involves an awareness of the enormous transition in social and emotional functioning that occurs during adolescence. This transition will most likely result in changes in the nature and functioning of the attachment system.

Additional findings support the idea that family environmental factors, such as attachment types and parental nurturing behaviors, have a role in the development of anger/hostility in children. More precisely, adolescents who defined themselves as insecurely attached demonstrated higher levels of anger/hostility than adolescents who classified themselves as securely attached. Further, low levels of parental warmth and affection and high levels of rejection, control, and inconsistency were marked by increased levels of anger/hostility [32].

Hence, Bowlby proposed that dysfunctional anger is at the center of insecure attachment, indicating that those who are insecurely attached experience a restlessness between their underlying desire for proximity and their expectations of others’ responsiveness [33]. As their behaviors compete with this intrinsic need, angry feelings and behaviors become prominent, consequently leading to an inappropriate expression of anger. Indeed, most recent studies uphold the association between insecure attachment style and anger expression patterns, thus supporting Bowlby’s ideas. Mikulincer and Shaver examined attachment style differences in anger outcomes [16]. They proposed that children in all four attachment categories experience anger and disappointment, yet it is how their caregiver reacts to their distress that, to some extent, decides how the child, at last, adapts to these feelings. Firstly, they found that Dismissive-Avoidant children deactivated distress, and in this manner, anger was communicated in more unintended ways. Secondly, Fearful-Avoidant children were persistently furious and expressed different feelings through their anger. Thirdly, Anxious-Preoccupied children were overwhelmed with an emotional overflow that they could detach or become aggressive toward themselves or others. Fourthly, secure children also lost control but were more likely to express it directly to other people. At the point when the source of anger is not available, they can depend on mental representations that concede to self-soothing and returning to a state of emotional well-being. Thus, insecure children lack a healthy mental depiction of soothing, leading to more rather than less anger [29, 30].

A relationship has been detected between insecure attachment and levels of general aggression [29, 30]. Similar associations have been found between attachment insecurity and hostility [28, 34]. As such, most evidence shows that either the dimension of attachment anxiety or insecure attachment groups characterized by high levels of attachment anxiety are especially correlated with higher levels of self-reported trait anger than those indicating attachment security [34, 35]. The idea that attachment styles can affect the external expression of anger in an individual educes a reason why people develop wrath issues and behavioral problems in adolescence, which escalate into adulthood [16]. Investigating the relationship between attachment styles and anger, aggression, and hostility among young adults would benefit psychology because of its in-depth examination of how the child-rearing environment influences growing. Anger manifestations can produce harmful outcomes on health and can influence the behavior of individuals who suffer from several mental health conditions; therefore, understanding bonding traits could offer insight into individuals at risk to prevent behavioral outbursts.

In Lebanon, a Middle Eastern country with a long history of conflicts and economic and political instability, epidemiology statistics on the attachment distribution in adolescents or children are scarce. Lebanon has many cultural similarities with the Arab world, but it also offers features that set it apart from many of its Arab neighbors. Lebanon's culture is relatively contemporary and matches some cultures in Southern Europe. However, this does not negate the fact that many regions remain inherently conservative and attached to traditions. Since people in Lebanon differ from other foreign countries in terms of cultural and religious backgrounds, and due to the key role that culture plays in determining the sense of self and others, the impact of cultural variations on self/other-construal may influence individual attachment figure [36]. Furthermore, Lebanese youths and adults confront mounting challenges and different stressors driving them to significant levels of rejection and destabilization, including security risks, sociopolitical instability, a shaky economy, limited work opportunities (especially for skilled youth), and internal insecurity from the Syrian crisis and the subsequent immigration [37]. In addition, a previous study showed that a secure attachment style was significantly associated with lower addiction to alcohol, cigarettes, and waterpipes, whereas insecure attachment was significantly associated with higher addiction to cigarettes, waterpipes, alcohol, and the internet among Lebanese adolescents [38]. All these factors can expand the risk of anger. Hence, identifying and apprehending attachment styles among Lebanese adolescents might offer a plausible future solution to controlling and limiting the persistent anger issues in the Lebanese population.

So far, no study has been carried out relating the three facets of anger to attachment styles in Lebanese adolescents. Lebanon lacks data on insecure attachment styles and the affective and cognitive aspects of anger and behavioral anger expression among Lebanese youth. Faced with the prospect of an independent Lebanese model of self and since previous international studies have shown a linkage between personal development, the basis of attachment style, on the one hand, and adolescents’ anger demonstrations, on the other hand, appraising the relation of these constructs in young adults who live in Lebanon was deemed necessary.

### The current study

While researchers have found a link between insecure attachment and anger expression and the fact that adolescents react to a perceived threat in several ways, including anger, aggression, and hostility, little is known about how these reactions can be interpreted based on the different attachment types, which may suggest different approaches for better control or regulation of anger. Specifically, the current study explored whether preoccupied, dismissing, fearful, and secure attachment styles serve as a bridge to anger-occurring problems. Therefore, this study aims to examine the relationship between attachment dimensions and anger expression (trait anger, hostility, physical aggression, and verbal aggression) among Lebanese adolescents.

**Fig. 1:**
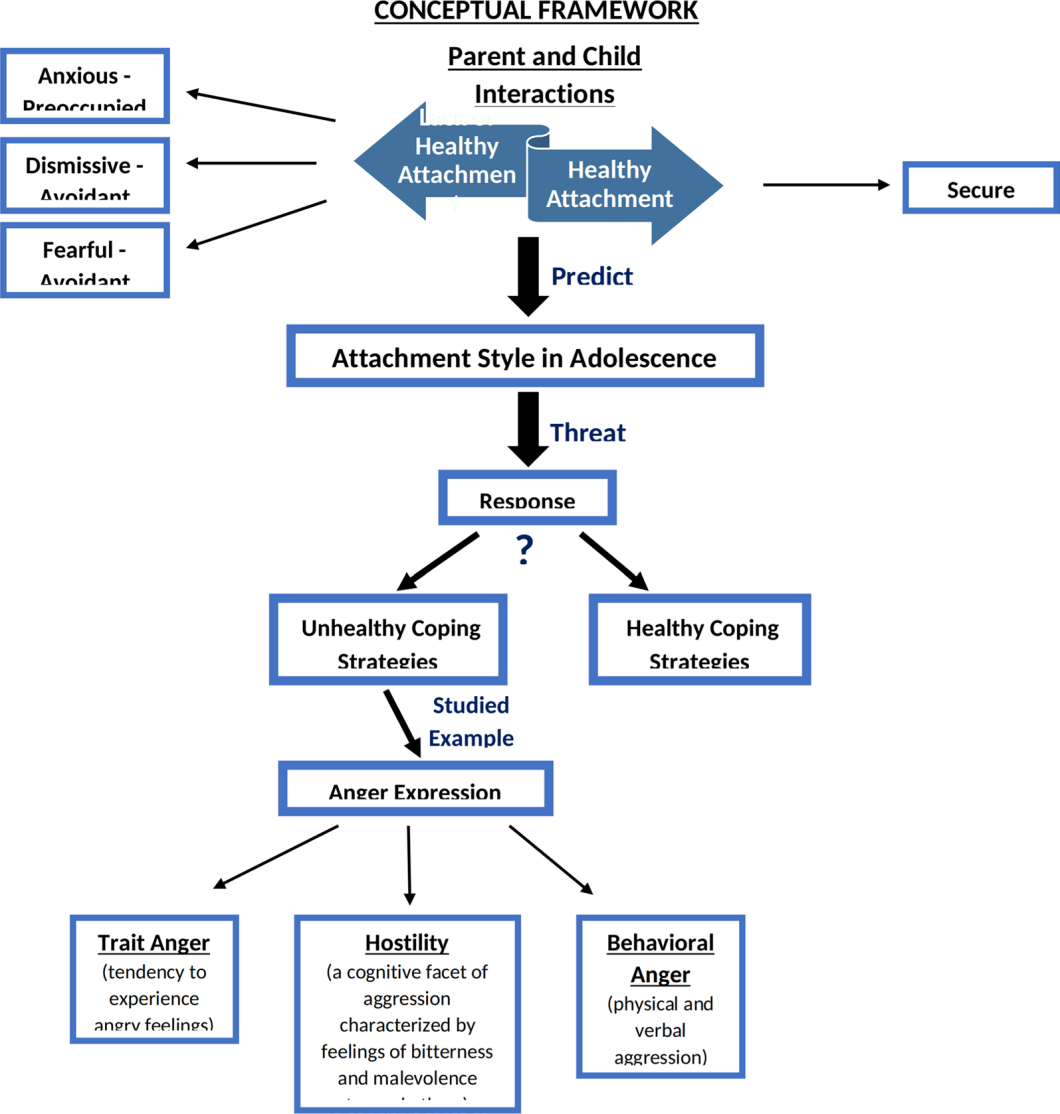
Conceptual framework of attachment theory and its connection with anger/aggression

This study highlights the importance of differentiating anger expression, and following the attachment theory, it was hypothesized that, compared to all insecure attachment styles, secure attachment style would be correlated with higher ratings of self-soothing and returning to a state of emotional well-being, thus experiencing anger more functionally. A different pattern was predicted for insecure attachment, i.e., the preoccupied, dismissing, and fearful styles. It was assumed that participants with an insecure attachment would endorse higher levels of anger, hostility, and verbal and physical aggression and manifest more signs of dysfunctional anger than their securely attached counterparts. Hence, the conceptual framework of attachment theory and its connections with anger/aggression/hostility was developed (Fig. 1).

## Methods

### Participants

This cross-sectional study was performed between January and May 2019 among Lebanese high-school students. A proportionate random sample of schools from all Lebanese Mohafazat (Beirut, Mount Lebanon, North, South, and Beqaa) was exerted as a recruitment method. This sample is based on an exhaustive list of schools obtained from the Ministry of Education and Higher Education in Lebanon. From each Mohafaza, a proportionate number to the total number of schools was selected; in case of school refusal, no replacement was made. Out of the 18 private schools contacted, 2 refused to participate, and the remaining 16 were located as follows: 4 in Beirut, 2 in South Lebanon, 6 in Mount Lebanon, 2 in North Lebanon, and 2 in Beqaa. All students aged 14–17, who were physically present on the day the survey was administered, were eligible. They were free to accept or refuse to participate, and no financial compensation was offered in return for their participation. Students who refused to fill out the questionnaire were excluded. Out of 2000 questionnaires distributed, 1810 (90.5%) were completed and collected back. The methodology used has been previously described [39–46].

The mean age was 15.42 ± 1.14 years (minimum of 14 and maximum of 17), with 53.3% females. In addition, the majority have their parents living together (88.1%), and 11.9% of the adolescents had separated/divorced parents. The mean number of siblings was 2.38 ± 1.71, and the mean household crowding index was 1.01 ± 0.64 (Table 1).

**Table 1:** Sociodemographic characteristics of the sample population (N = 1810)

	Frequency (%)
*Gender*	
Male	844 (46.7%)
Female	963 (53.3%)
*Parents’ status*	
Living together	1581 (88.1%)
Separate	213 (11.9%)

	Mean ± SD
Age (years)	15.42 ± 1.14
Number of siblings	2.38 ± 1.71
Household crowding index	1.01 ± 0.64
Secure attachment style	3.35 ± 1.80
Fearful attachment style	3.38 ± 1.78
Preoccupied attachment style	4.56 ± 1.71
Dismissing attachment style	4.23 ± 1.84
Physical aggression score	30.54 ± 8.84
Verbal aggression score	16.45 ± 6.30
Anger score	24.53 ± 6.96
Hostility score	28.10 ± 8.76

### Minimal sample size calculation

The G-power software estimated a minimum number of 395 students, taking an effect size f2 = 2%, α = 5%, β = 80%, and 10 factors to be considered in the multivariable analysis.

### Procedure

The self-administered questionnaire took approximately 60 min to be completed. Students were asked to fill it out in class to prevent their parents’ influence when answering the questions. A member of the research team was present in the classroom to clarify questions to students when necessary. The completed questionnaires were gathered back in closed boxes at the end of the process and submitted for data entry. The anonymity of the participants was guaranteed during the data collection process.

### Measures/instruments

The questionnaire used during the interview was in Arabic, the native language of Lebanon. The first part assessed the sociodemographic details of the participants (i.e., age, gender, smoking status, parents’ status). The household crowding index was calculated by dividing the number of persons living in the house and the number of rooms in the house, excluding the bathroom and the kitchen [47]. The Total Physical Activity Index was used to evaluate the physical activity during leisure time. It assessed the intensity, duration, and frequency of performing an activity. The total score was computed by multiplying the intensity, duration, and frequency of daily activity [48]. The second part of the questionnaire included the following scales:

#### Relationship Questionnaire (RQ)

This self-assessment measure of attachment behavior [49] consists of four subscales corresponding to the four adult attachment styles: Style A (secure), Style B (fearful), Style C (preoccupied), and Style D (dismissing). These subscales define facets of subjects' relationships and attachment patterns. The Secure subscale assesses comfort with being close to and relying on others ("I find it easy to get emotionally close to others"). The Fearful subscale tracks fear over being hurt by others in relationships ("I find it difficult to trust others completely"). The Preoccupied subscale measures the extent to which participants are concerned that people do not care for them ("I find that people don't want to get as close as I would like"). The Dismissing subscale tests the lack of desire for emotional closeness with others ("I prefer not to depend on people"). Each subscale is rated on a 7-point scale from 1 (being not at all like me) to 7 (being very much like me), with higher scores indicating higher attachment style. The Cronbach’s alpha for this scale was 0.970.

This measure was used to assess attachment types in our population, in the absence of a specific instrument for children/adolescents.

#### The Buss-Perry Aggression Questionnaire (BPAQ)

The Buss-Perry Aggression Questionnaire (BPAQ) is a 29-item instrument rated on a 5-point Likert scale from 1 (extremely uncharacteristic of me) to 5 (extremely characteristic of me). The BPAQ includes four subscales: Physical Aggression (PA, items 1–9), for example, “Once in a while I can’t control the urge to strike another person” item 1, Verbal Aggression (VA, items 10–14), for instance, “I tell my friends openly when I disagree with them” illustrates item 10, Anger (A, items 15–21), for example, “I flare up quickly but get over it quickly” depicts item 15, and Hostility (H, items 22–29), for instance, “I am sometimes eaten up with jealousy” illustrates item 22 [50]. The score of each subscale is the sum of the ratings for its items. The two items (7 and 18) worded in the direction opposite to aggression are reverse-scored. The total score for aggression is the sum of the four subscale scores. Higher scores indicate higher aggressive behavior. The Cronbach’s alpha values were as follows: Total scale (0.846), physical aggression (0.565), verbal aggression (0.586), anger (0.402), and hostility (0.625).

### Translation procedure

The forward and backward translation method was carried out for all scales by two independent psychologists. All translators were informed of the intent of the study before translation. The back-translated English questionnaire was compared to the original English questionnaire by the principal investigator to detect discrepancies and correct inconsistencies between the two versions. Revisions of problematic questions were communicated to the translators in charge of updating the version. The forward–backward translation process was repeated until all ambiguities were resolved [51–57].

### Statistical analysis

SPSS software version 23 was used to perform data analysis. Cronbach’s alpha values were recorded for reliability analysis for all the scales. Missing values were not replaced since they constituted less than 10% of the entire database. The sample was normally distributed as verified by visual inspection of the histogram, while the skewness and kurtosis were below |1.96| [58]. Pearson correlation was used for the linear correlation between the aggression domains and the attachment styles.

A multivariate analysis of covariance (MANCOVA) was carried out to compare multiple measures (each scale was taken as a dependent variable) between the different attachment styles, taking into account potential confounding variables, i.e., age, gender, house crowding index, number of siblings, smoking status, and physical activity score. Attachment styles were divided into high and low attachment based on the median, in the absence of cut-off values for these scores. The categories of attachment styles were considered as independent variables in the model to compare the mean of aggression between high and low levels. A p value less than 0.05 was considered significant.

## Results

### Association between aggression and attachment styles

A significant positive correlation was found between the secure attachment style and all the aggression scores. Also, the fearful attachment style was positively associated with all the aggression except the hostility score. The preoccupied and dismissing styles were negatively correlated with all the aggression domains (Table 2).

**Table 2:** Correlation between the aggression scale and attachment styles

	Physical aggression score		Verbal aggression score		Anger score		Hostility score	
	Correlation coefficient	p value	Correlation coefficient	p value	Correlation coefficient	p value	Correlation coefficient	p value
Secure attachment style	0.071	**0.008**	0.069	**0.010**	0.174	** < 0.001**	0.167	** < 0.001**
Fearful attachment style	0.064	**0.019**	0.068	**0.012**	0.141	** < 0.001**	− 0.008	0.774
Preoccupied attachment style	− 0.115	** < 0.001**	− 0.171	** < 0.001**	− 0.236	** < 0.001**	− 0.067	**0.014**
Dismissing attachment style	− 0.084	**0.002**	− 0.265	** < 0.001**	− 0.108	** < 0.001**	− 0.149	** < 0.001**

Numbers in bold indicate significant *p*-values.

### Multivariate analysis

The MANCOVA analysis was performed, taking the scales as the dependent variables and the adult attachment styles as the independent variable, adjusting for the covariates (age, gender, house-crowding index, number of siblings, smoking status, and physical activity score).

There was a statistically significant difference between the secure attachment style (F(4, 1287) = 31.42, p < 0.001, partial η2 = 0.089), fearful (F(4, 1287) = 7.043, p < 0.001, partial η2 = 0.021), preoccupied (F(4, 1287) = 4.22, p < 0.001, partial η2 = 0.013), and dismissing (F(4, 1287) = 16.70, p < 0.001, partial η2 = 0.049) attachment styles on the combined aggressions dependent variables after controlling for age, gender, house crowding index, number of siblings, smoking status, and physical activity score.

The secure attachment style was significantly associated with physical aggression, verbal aggression, and anger. The fearful attachment style was significantly associated with physical and verbal aggression. The preoccupied style was significantly associated with verbal aggression; however, dismissing attachment was significantly associated with physical aggression and anger (Table 3).

**Table 3:** Multivariate analysis of covariance (MANCOVA)

	Beta	p value	Effect size (*n*^*2*^)	95% Confidence interval	
				Lower bound	Upper bound
*Physical aggression score*					
Age	− 0.237	0.233	0.001	− 0.626	0.153
Gender	− 0.067	0.886	0.0001	− 0.982	0.848
Number of siblings	− 0.465	**0.006**	0.006	− 0.799	− 0.132
Smoking status	− 2.701	** < 0.001**	0.015	− 3.894	− 1.508
Physical activity	0.108	** < 0.001**	0.034	0.076	0.139
Household crowding index	2.965	** < 0.001**	0.050	2.261	3.670
Secure attachment style (high vs low*)	− 2.707	** < 0.001**	0.018	− 3.796	− 1.618
Fearful attachment style (high vs low*)	2.601	** < 0.001**	0.014	1.393	3.810
Preoccupied attachment style (high vs low*)	0.222	0.721	0.0001	− 0.998	1.442
Dismissing attachment style (high vs low*)	1.817	**0.001**	0.009	0.750	2.884
*Verbal aggression score*					
Age	− 0.078	0.586	0.0001	− 0.360	0.204
Gender	0.903	**0.008**	0.006	0.241	1.566
Number of siblings	0.167	0.174	0.001	− 0.074	0.409
Smoking status	− 2.237	** < 0.001**	0.020	− 3.101	− 1.373
Physical activity	0.071	** < 0.001**	0.029	0.049	0.094
Household crowding index	2.301	** < 0.001**	0.057	1.790	2.811
Secure attachment style (high vs low*)	− 2.172	** < 0.001**	0.022	− 2.960	− 1.384
Fearful attachment style (high vs low*)	1.238	**0.006**	0.006	0.363	2.113
Preoccupied attachment style (high vs low*)	− 0.926	**0.040**	0.003	− 1.809	− 0.043
Dismissing attachment style (high vs low*)	− 0.161	0.682	0.001	− 0.934	0.611
*Anger score*					
Age	− 0.317	0.054	0.003	− 0.639	0.005
Gender	− 0.582	0.132	0.002	− 1.339	0.175
Number of siblings	0.166	0.237	0.001	− 0.110	0.442
Smoking status	− 3.361	** < 0.001**	0.033	− 4.348	− 2.374
Physical activity	0.094	** < 0.001**	0.038	0.068	0.120
Household crowding index	0.370	0.214	0.001	− 0.213	0.953
Secure attachment style (high vs low*)	1.931	** < 0.001**	0.014	1.031	2.832
Fearful attachment style (high vs low*)	0.866	0.090	0.002	− 0.134	1.866
Preoccupied attachment style (high vs low*)	0.295	0.567	0.001	− 0.715	1.304
Dismissing attachment style (high vs low*)	2.084	** < 0.001**	0.016	1.201	2.967
*Hostility score*					
Age	− 1.061	** < 0.001**	0.022	− 1.448	− 0.674
Gender	0.611	0.188	0.001	− 0.298	1.520
Number of siblings	0.411	**0.015**	0.005	0.079	0.742
Smoking status	− 4.927	** < 0.001**	0.049	− 6.113	− 3.742
Physical activity	0.054	**0.001**	0.009	0.022	0.085
Household crowding index	3.226	** < 0.001**	0.060	2.526	3.926
Secure attachment style (high vs low*)	0.042	0.939	0.0001	− 1.040	1.124
Fearful attachment style (high vs low*)	0.215	0.726	0.001	− 0.986	1.416
Preoccupied attachment style (high vs low*)	1.170	0.058	0.003	− 0.042	2.382
Dismissing attachment style (high vs low*)	− 0.372	0.491	0.001	− 1.432	0.688

Numbers in bold indicate significant *p*-values

In the global model, the independent variable is the adult attachment styles. Covariates are: age, gender, house crowding index, number of siblings, smoking status, and physical activity score

^*^Reference group

### Association between aggression and attachment styles adjusted for age, gender, house-crowding index, number of siblings, smoking status, and physical activity score

**Fig. 2:**
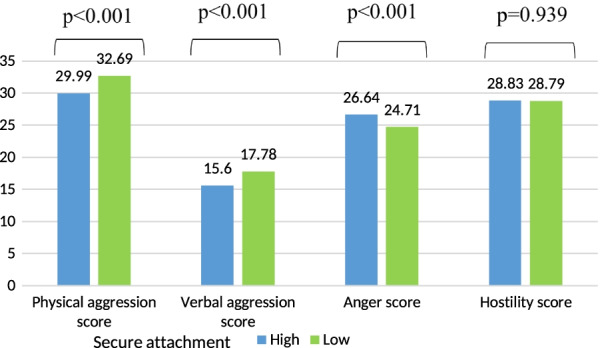
Mean values of the aggression scores according to the secure attachment adjusted for age, gender, house-crowding index, number of siblings, smoking status, physical activity score

After adjusting for all covariates, higher means of physical and verbal aggression scores were significantly found in participants having a low secure attachment style as compared to those with a high level. However, a high mean of anger score was found in participants with a high secure attachment as compared to low (Fig. 2).

**Fig. 3:**
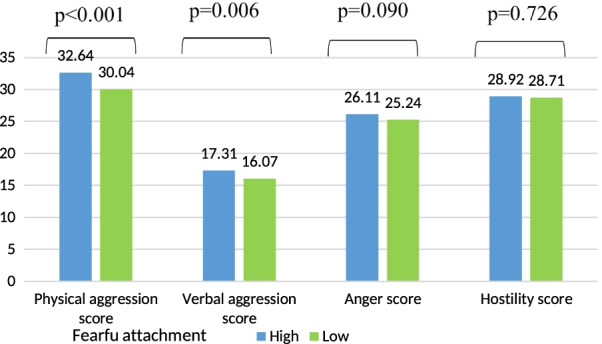
Mean values of the verbal aggression scores according to the fearful attachment adjusted for age, gender, house-crowding index, number of siblings, smoking status, physical activity score

Higher mean physical and verbal aggression were significantly found in participants having a high fearful attachment style as compared to low level (Fig. 3).

**Fig. 4:**
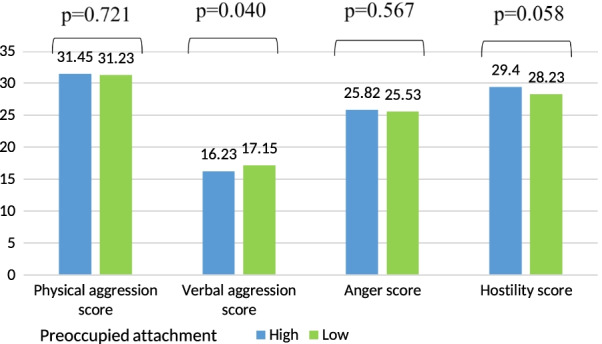
Mean values of the aggression scores according to the preoccupied attachment adjusted for age, gender, house-crowding index, number of siblings, smoking status, physical activity score

**Fig. 5:**
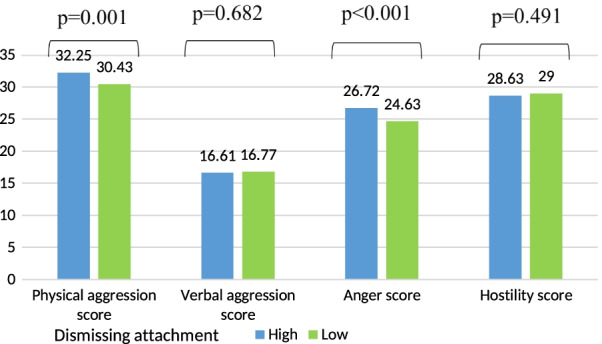
Mean values of the aggression scores according to the dismissing attachment adjusted for age, gender, house-crowding index, number of siblings, smoking status, physical activity score

Lower mean verbal aggression score was significantly found in participants having a preoccupied attachment style (Fig. 4). Higher mean physical aggression and anger scores were significantly found in participants having a high dismissing style as compared to a low level (Fig. 5).

## Discussion

To our knowledge, this study is the first to examine how attachment styles, as measured by the Relationship Questionnaire, are a critical component in determining the way anger is expressed among Lebanese youth. As predicted, the results indicate attachment-related differences in anger expression (hostility, anger, physical aggression, and verbal aggression) among adolescents, with insecure individuals being highly reactive to anger.

Overall, individuals with a secure attachment style scored lower in anger expression than those with insecure attachment. It has been referred that the ability of parents and children to communicate with each other is related to a secure attachment [59]. Our results converge with previous findings showing that adolescents who have positive communication with their parents are less inclined to engage in aggressive tendencies and risky behaviors [59]. Furthermore, the nature of early attachments affects not only the individual’s emerging self-concept and view of the social world [17] but also their social capacities, sense of well-being, and subsequent relationships [60].

Higher physical and verbal aggression scores were detected among adolescents with insecure attachments. Previous reports showed that the emergence of some aggressive behaviors was strongly related to communication with parents [61], where better communication between parents and teens induces a higher feeling of security and an active exchange with others throughout life. In other words, those with high insecurity may struggle to limit their anger to constructive forms. However, people with these insecurities are more likely that engage in aggressive behaviors [62].

In our study, a fearful attachment was a significant independent predictor of anger score. Previous findings revealed the potential deleterious outcomes associated with insecure attachment styles, particularly the fearful type [63]. It has been shown that individuals with a negative model of the self and high anxiety about rejection exaggerate the negative consequences of conflict and react with excessive anger and hurt [63]. Indeed, fearful attachment is built on an image of the self as undeserving of affection from others and an image of caretakers as untrustworthy and even threatening. Research shows that fearfully attached individuals share a history of persistent rejection by caregivers [49]. Due to this lack of bonding, fearfully attached young adults tend to be more vulnerable to anger than secure or preoccupied individuals, who usually have more positive expectations of caretakers.

Our results showed a significant inverse relationship between secure attachment style and anger score. In line with previous research that explored the effects of attachment styles on anger and parental attachment, dysfunctional anger was associated with an insecure attachment [64]. Another study investigated the functional nature of anger among secure adolescents by enrolling adolescent participants in a frustrating problem-solving assignment with the aid of a friend and decrypted their dissatisfaction and anger during the task and their negative behavior toward the friend (e.g., discarding/ignoring the friend’s suggestions and ideas without discussion). Dissatisfaction and anger were correlated with more aggressive behavior only in the case of insecure adolescents [65].

Besides, higher hostility scores were found with preoccupied attachment style. This result is consistent with the relatively small body of existing research evaluating the association of anxious attachment with anger and hostility, which inferred that anxious attachment is correlated with irritability, anger, and hostility [29, 66]. Additionally, people with a negative view of others (i.e., dismissing attachment style) scored high on hostility in our study. Several studies have pointed out this association. Compared to non-hostile individuals, those high in hostility were reported with more interpersonal conflicts [67] and more negative and fewer positive interpersonal interactions [68]. They were less able to recognize positive responses from others [69]. The hostility-dismissing attachment style relationship could be explained by the fact that hostility is thought to reflect a mistrust and suspicion of others [3].

A strong negative relationship was shown between hostility scores and the age of participants, consistent with previous findings and confirming the correlation between hostility levels and character maturity [70]. Previous research has also shown that interpersonal trust increases as people grow old [71].

In this study, anger expression scores were significantly higher among adolescents with a high physical activity index. It is noteworthy that most adolescents are active but probably unaware of the beneficial effect of low-intensity exercise on managing anger and decreasing the likelihood of aggressive outbursts compared with high-intensity activities [13]. Overall, exercising is seen as a mood enhancer, but high-intensity exercise has been found to cause additional tension and stress physically and mentally [13]. Previous findings revealed that the increased duration and consistency of exercise are beneficial in enhancing the mood and reducing one’s overall anger. However, high-intensity exercise has created more stress, thus producing increased anger reactions [13].

This study could demonstrate that an overcrowded household can lead to increased levels of anger expression. Previous findings supported the notion that a higher household crowding index negatively affects marital and family relations (i.e., marital instability and more marital argument, parent–child tension), the core principle of attachment styles [72]. Additionally, this positive match between crowded living conditions and anger expression might be due to the negative influence of overcrowding aspects, such as increased physical contact, lack of sleep, and lack of privacy, on family relationships [73].

Consistent with previous research, it was hypothesized that attachment styles contribute to individual differences in anger expression tendencies. Our results provide a preliminary contribution to research and practice, indicating the need for psychological support in adolescents and longitudinal studies to confirm our findings. Moreover, the data could be included in a developmental framework. Adolescents have quite limited interpersonal experiences. As these young adults evolve and encounter more extensive social interactions, their specific attachment styles may be altered. It would be interesting to track them through and after their college years to categorize their experiences and, maybe, their modified attachment styles that could follow their growth and development, confirm the cross-time correlation between age and hostility scores, and possibly unveil unseen relationships between age and other scores.

Furthermore, anger may be more prevalent in certain groups of adolescents, or it may be directed more towards specific figures, such as siblings, or in some cases, the adolescent may not have been allowed to experience or express anger. For instance, if the adolescent grew up in a family that shamed or condemned emotional expression, or if the adolescent grew up in a home with an abusive parent, the adolescent may associate anger with fear, danger, or damaged relationships, leading to a more negative perception of the relationship with parents and siblings. In this regard, future studies on the role of multiple placements, such as unstable living conditions, greater attachment to siblings, adoption, frequent school changes, and difficulties, are needed in this area. Moreover, while there are various variations between genders, anger is a natural feeling shared by both. Men have a reputation for being more prone to anger, despite evidence showing women are more emotionally expressive. Further studies focusing on gender disparities are necessary to explore such assumptions.

Our findings suggest that preventive measures should be adopted to limit the possible progression of anger symptoms. When anger becomes uncontrollable, it can lead to all types of problems, including erratic behavior, assault, abuse, addictions, and troubles with the law. In these cases, anger hinders individual decision-making, damages relationships, and otherwise causes harm. Recognizing warning signs of anger is particularly relevant to controlling anger and handling triggers but without suppressing and storing it up, thereby limiting emotional damage. Anger management methods involve relaxation techniques, monitored breathing exercises, cognitive behavioral therapy and imagery, problem-solving, and enhancing communication strategies and interpersonal skills.

### Limitations

While offering a relevant analysis of the relationship between attachment styles and anger expression, the current paper is not without limitations. The results could not demonstrate that the gender of participants was a significant moderator of the link between both attachment dimensions and anger expression scores. Moreover, negative emotions, including bad mood, tiredness, pain, and frustration, particularly when accompanied by high arousal, and hurtful events, may produce aggression. Therefore, the sample characteristics did not account for these covariances in the examined relationship. Besides, anger has a strong genetic basis, which has not been reported. Another drawback of this study is its cross-sectional research design. Although the results were acquired from adolescents at a single point in time, the findings do not fully reflect the complexities of the attachment system over time. Moreover, all variables were evaluated through a self-reported questionnaire and not a clinical interview by a healthcare professional; thus, the responses might lack precision and accuracy and may have been subject to reporting bias. In addition, the Buss-Perry Scale and RQ scales were not validated in Lebanon. Participants’ language skills were not controlled for any different cultural backgrounds. Lastly, due to the selection process of schools, there may be a selection bias since public schools were not considered; therefore, the study results might not be generalizable.

## Conclusion

Our findings revealed a significant relationship between both insecure attachment dimensions and the tripartite model of anger expression. This study adds to the anger literature by providing a more informed understanding of how variations in anger expression are linked to the processing of interpersonal interactions, the hidden facets of attachment systems. Insecure adolescents were more prone to high levels of anger expression (verbal and physical aggression, anger, hostility). Intense physical activity and a crowded household increased anger expression scores. Also, older age decreased hostile attitudes in our sample.

In addition to the interesting results, this study generated perplexing questions. It would be interesting to look if certain attachment styles attract anger from others as well. More importantly, the results emphasized the need for future studies to give further insights into whether anger management interventions should concentrate on creating constructive attachment models rather than solely relying on anger control techniques. However, the attachment style is a personal characteristic that appears to linger throughout life and may be difficult to alter. It might also be more efficient to emphasize potentially modifiable factors other than physical activity already assessed in this study, such as psychosocial constructs, the critical components in shaping people’s traits, as a way to extend the chances of anger management.

## Data Availability

All data generated or analyzed during this study are not publicly available to maintain the privacy of the individuals’ identities. The dataset supporting the conclusions is available upon request to the corresponding author.
